# Arthritis alleviation: unveiling the potential in *Abrus precatorius* macerated oil

**DOI:** 10.2144/fsoa-2023-0248

**Published:** 2024-05-15

**Authors:** Sukanya Vijayan, Thirumal Margesan

**Affiliations:** 1 Department of Pharmacognosy, SRM College of Pharmacy, SRM Institute of Science & Technology, Kattankulathur, 603203, Chengalpattu, Tamil Nadu, India

**Keywords:** *Abrus precatorius*, anti-arthritic, antioxidant, macerated oil, molecular docking

## Abstract

**Aim:** This study endeavors to explore the anti-arthritic effects of macerated oil derived from the plant's aerial parts. **Methods:** The macerated oil was prepared using maceration in coconut oil, and its phytochemical composition was elucidated using GC-MS. To assess its anti-arthritic activity, *in-vitro* studies were conducted using various assays. **Results & conclusion:** The macerated oil showed better antioxidant and anti-arthritic potential by *in*-*vitro* investigations. Molecular docking studies elucidated potential binding interactions between specific constituents of the oil and critical molecular targets implicated in the pathogenesis of arthritis, further substantiating its therapeutic potential. The results demonstrated that *Abrus precatorius* macerated oil could ameliorate arthritis severity in a dose-dependent manner.

## Plain language summary

The study explores whether the oil from the *Abrus precatorius* plant can help people with arthritis. Arthritis is a painful condition that causes swelling and stiffness in the joints. Researchers are curious if this plant oil can ease the pain and reduce inflammation associated with arthritis. They are conducting experiments to see if it works and is safe to use. In the research, we study the effects of this plant oil on arthritis symptoms. This oil is obtained by soaking *Abrus precatorius* plant parts in coconut oil. Our results could offer a new and natural way to alleviate arthritis symptoms for those who suffer from it. This would be a significant finding as it might provide a less invasive and potentially more accessible option for managing arthritis discomfort. However, it is important to remember that more research is needed to ensure the oil is both effective and safe for arthritis relief.

## Abstract (plain language summary)

Arthritis is a prevalent and debilitating global issue, causing pain, disability and reduced quality of life.

Effective and safe arthritis treatments remain a significant challenge.

This study explores the potential of macerated oil from the *Abrus precatorius* plant as a novel arthritis therapeutic agent.

The macerated oil was created by soaking the plant's aerial parts in coconut oil.

Gas chromatography-mass spectrometry (GC-MS) was used to analyze the chemical composition of the oil.

*In-vitro* studies were conducted to evaluate the oil's anti-arthritic properties using relevant experimental models.

Molecular docking studies identified potential interactions between oil components and molecular targets linked to arthritis development.

The study's results demonstrated that *Abrus precatorius* macerated oil effectively reduced arthritis severity in a dose-dependent manner.

This suggests the oil's potential as a safe and efficient treatment for arthritis, offering hope for better arthritis management.

Further research and clinical trials are required to confirm and fully validate the therapeutic benefits of this plant-derived oil.

Rheumatoid arthritis (RA) is a complex autoimmune disorder that predominantly affects joints. It is characterized by the expansion of synovial tissue, the development of pannus, the degradation of cartilage and the occurrence of systemic complications [1]. It is the most prevalent form of inflammatory arthritis, leading to significant morbidity and mortality rates [2]. Studies show that RA and sex are strongly correlated, with women three-times as likely than males to get the condition. The frequency rises gradually with age, with women three-times more often than men [3]. Variations between genders have been documented regarding the progression and features of the disease, impacting both patients' reported outcome measures and their perception of pain [4]. The increased prevalence of RA in women compared with men has been attributed to a combination of genetic, hormonal and environmental factors [5]. Decreasing levels of estrogen and/or progesterone during menopause and postpartum may raise the likelihood and intensity of RA in women [6]. RA is an intricate condition shaped by a combination of genetic and epigenetic factors, alongside environmental elements such as cigarette smoke, dust exposure, silica exposure and the microbiome [7]. The likelihood of developing RA appears to be influenced by daily habits. Key areas of research in this context include smoking, dietary patterns and body weight. Among these factors, exposure to nicotine, particularly through smoking, stands out as the most widely recognized environmental risk for RA [8,9]. Living in polluted areas also increases the risk of RA and producing RA-specific autoantibodies, as it triggers innate immune responses and adaptive immune responses [10]. Genetic risk factors for RA, including human leukocyte antigen gene variations, account for 60% of the disease risk, affecting the immune system's ability to distinguish cells from foreign invaders [11]. Symptoms of the RA can vary and can be mild or severe. Frequently observed indications encompass discomfort in the joints, along with tenderness, swelling and a sense of rigidity. This typically impacts smaller joints. Morning stiffness, fatigue, weakness, fever, loss of appetite and weight loss can also occur. RA can cause problems in other parts of the body [12].

RA is marked by the persistent inflammation of the synovium, which is the membrane lining the joints. This inflammation leads to the thickening of the synovium and the formation of pannus, a tissue that can erode cartilage and bone. The synovium is crucial for cartilage nutrient supply and joint lubricant production, forming the structural framework of the synovial interstitium [13]. The destruction of cartilage and bone leads to joint pain, stiffness and swelling [14]. During the initial phase of the disease, T lymphocytes and B cells get activated from external stimuli and stimulate macrophages and fibroblasts to produce cytokines like IFN-γ, TNF-α, IL-1, IL-6 and IL-15 [15]. Cytokines like IL1 and TNF-α stimulate the production of reactive oxygen and nitrogen species generation, increase chondrocyte degradation pathways and matrix breakdown, while also inhibiting new cartilage formation [16]. These cytokines induce other cytokine synthesis, upregulate adhesion molecules, activate osteoclasts and other inflammatory mediators. They also induce the acute phase response, systemic symptoms and B-cell activation [17]. RA is additionally distinguished by the existence of disease-specific autoantibodies known as rheumatoid factors, and anti-citrullinated peptide antibodies (ACPA), which encompass anti-CCP. These autoantibodies play a crucial role in defining the various disease patterns and characteristics associated with RA [18]. Intracellular signaling pathways, such as the mitogen-activated protein kinase (MAPK), JAK-STAT (Janus kinase/signal transducers and activators of transcription) and nuclear factor kappa-light-chain-enhancer of activated B cells (NF-κB) pathways, exert a pivotal influence on the onset of a broad spectrum of illnesses [19]. *In silico* studies, including molecular docking, allow researchers to predict how specific compounds interact with biological targets at the molecular level. This aids in understanding the potential mechanisms through which the identified constituents in the macerated oil may exert their anti-arthritic effects. Tumor necrosis factor-alpha (TNF-α) and interleukin-6 (IL-6) are cytokines that play pivotal roles in inflammatory processes, including those associated with arthritis. Targeting these specific molecules is of great interest in developing anti-arthritic interventions. Molecular docking studies help in identifying whether the constituents from the macerated oil could interact with and potentially modulate the activity of TNF-α and IL-6. By focusing on specific targets like TNF-α and IL-6, researchers can prioritize compounds from the macerated oil that show the most favorable interactions. This helps in selecting key bioactive components for further experimental validation. Molecular docking is an integral part of rational drug design. Understanding how compounds interact with specific targets provides valuable insights for designing new drugs or optimizing existing ones. This can potentially lead to the development of novel anti-arthritic agents derived from natural sources like *Abrus precatorius*.

Early diagnosis of RA is challenging due to its resemblance to other diseases and lack of a single blood test or physical finding [20]. The treatment strategy for RA is contingent upon the severity of the disease and an individual's response to therapy. The objective is to alleviate pain, diminish inflammation, hinder the progression of the condition and protect against joint damage [21]. Medications used in the treatment include ibuprofen, naproxen, diclofenac reduces pain and inflammation, while methotrexate, leflunomide, hydroxychloroquine, sulfasalazine, azathioprine slow RA progression but have side effects like nausea and liver damage. Biologic drugs, a newer treatment, can control RA but are expensive and have serious side effects like infections and cancer [22]. The need for safe and cost-effective alternatives to control the progression of RA arises from the ineffectiveness of anti-arthritis drugs and their extensive adverse effects [23]. Herbal medicines are promising contenders in the quest for the development of safe and effective arthritis treatments. This is because of their long-standing use in traditional practices, widespread availability and cost–effectiveness when contrasted with allopathic medications. Additionally, herbal drugs have shown promising results in reducing inflammation and pain associated with arthritis. Moreover, their natural composition and lack of severe side effects make them a favorable option for long-term use [24]. Therefore, further research and development of herbal drugs could potentially provide a breakthrough in the treatment of arthritis, offering patients a safer and more affordable alternative to traditional anti-arthritis drugs.

*Abrus precatorius*, also known as jequirity bean/rosary pea is a legume plant native to tropical and subtropical regions globally, renowned for its distinctive red seeds with a black spot [25]. The plant is a slender vine with oval-shaped leaflets, can grow up to 10 meters tall, produces small, white flowers in clusters. The seeds contain abrin, a potent protein similar to ricin found in castor beans, which can cause severe poisoning and death if ingested. For many generations, the plant has a long-standing history of application in traditional healing practices, especially for its leaves. Its historical application spans a diverse spectrum of health issues, encompassing ailments such as coughs, colds, fevers, malaria, jaundice, sprains, edema, arthritis, snakebites and an array of skin disorders [26]. This could be attributed to the existence of diverse phytochemical compounds like alkaloids, flavanoids, terpenoids, saponins present in the plant. In India, the paste of leaves of the plant has been used to treat muscle sprains [27]. The leaves are also used to treat sharp internal body pains in South Africa [28]. There is some evidence that the rosary pea may have anti-inflammatory and analgesic properties, which could be beneficial in the treatment of arthritis. Many plants belonging to the Fabaceae family have been reported to possess anti-arthritic activity [29]. Numerous traditional claims suggest the plant's efficacy in treating arthritis [30,31]. The traditional method involves boiling fresh leaves and using the resultant mixture externally for arthritis pain relief [32]. This study aims to investigate the potential anti-inflammatory effects of *Abrus precatorius* macerated leaf oil in coconut oil, as this may provide a more accessible and convenient form of application. While there is a wealth of anecdotal evidence supporting the use of *Abrus precatorius* for arthritis, scientific research on its efficacy is limited. Further studies are needed to validate its traditional uses.

## Materials & methods

### Plant material collection & taxonomic identification

**Figure 1.**
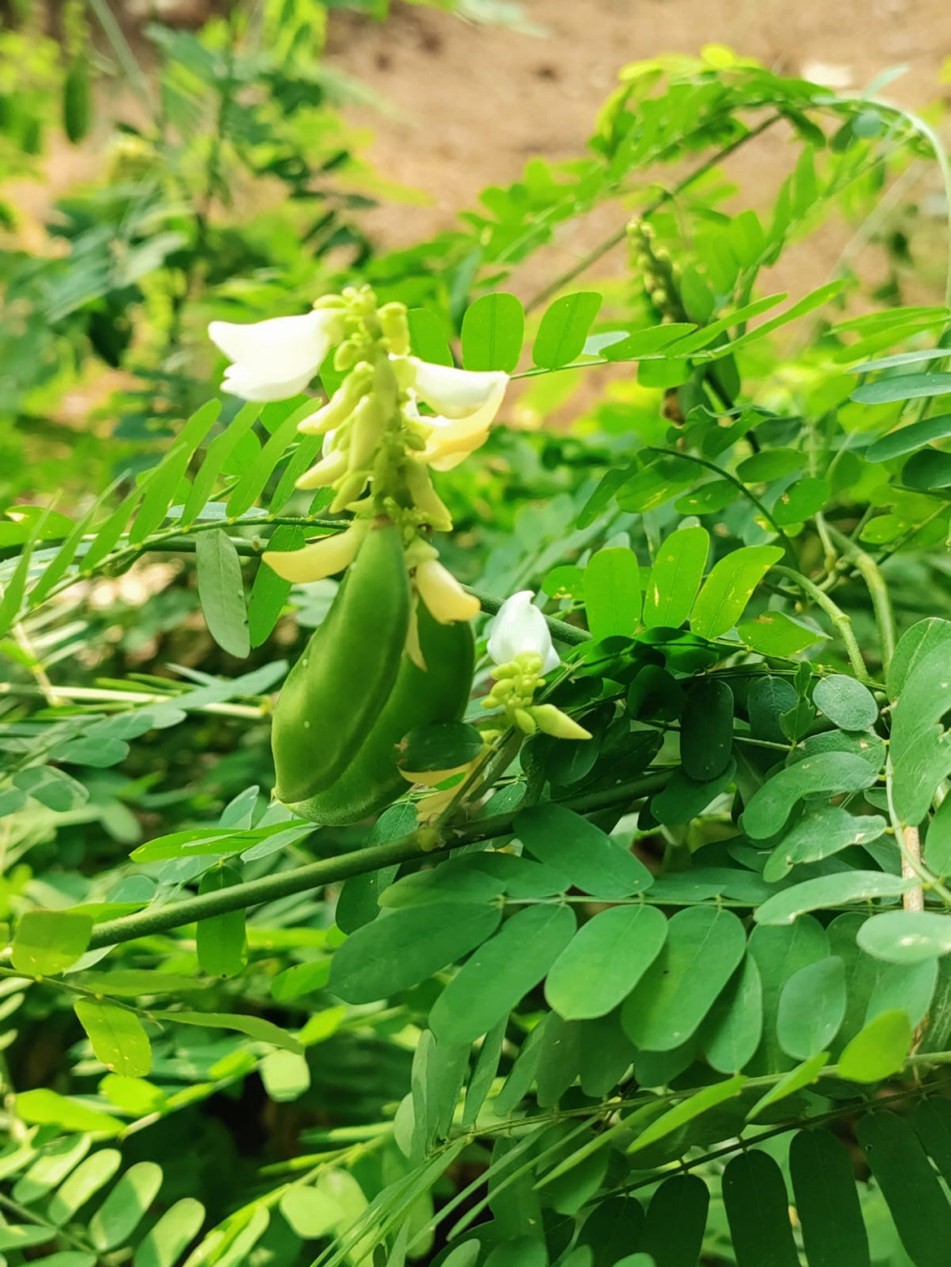
*Abrus precatorius* plant.

In August 2022, we procured the leaves of mature upper portions of *Abrus precatorius* from Palakkad, Kerala. To verify its authenticity, Dr KN Sunil Kumar, Research Officer and Head of the Department of Pharmacognosy at the Siddha Central Research Institute in Tambaram, Chennai, authenticated the specimen. The specimen has been assigned the registration number PCOG002-ACF. The image of *Abrus precatorius* plant is represented in Figure 1, respectively.

### Processing & preservation of plant material

The plant material (50 g leaves) underwent a process of cleaning, followed by gentle shade drying and subsequent coarse powdering through a no. 40 mesh. The resulting substance was then securely stored in an airtight container for future utilization.

### Formulating macerated oil

Macerated oil was prepared by maceration of 50 g of the powdered drug in 250 ml of coconut oil, and it is kept exposed in sunlight for about 40 days allowing for the optimum extraction of phytoconstituents from the plant [33,34]. The extracts then obtained were filtered and stored in an air tight container.

### Qualitative phytochemical analysis

The study determined the presence of various primary and secondary metabolites through standard method detection tests for alkaloids, tannins, phenols, saponins, flavonoids and glycosides [35].

#### Total phenolic content

Phenolic compounds possess antioxidant properties and have been associated with anti-inflammatory effects. The TPC analysis helps quantify the total amount of phenolic compounds present in the sample, providing insights into its potential antioxidant and anti-inflammatory capacities. The total phenolic content was determined using the Folin Ciocalteu's method [36,37]. For this, 1 ml samples and standard gallic acid solutions (0.007–1 mg/ml) were introduced in to individual test tubes. Then, 5 ml of distilled water and 0.5 ml of Folin Ciocalteu's reagent were mixed, followed by a 5-min incubation. Afterward, 1.5 ml of a 20% sodium carbonate solution was added, and the volume was adjusted to 10 ml with distilled water. The solution incubated for 2 h at room temperature, resulting in an intense blue color. Absorbance was measured at 750 nm using a UV-visible spectrophotometer after incubation. This process was repeated three times. A reagent blank with solvent served as a control, and gallic acid was the standard. The calibration curve was created using the standard gallic acid solution samples was expressed in milligrams equivalent to the weight of gallic acid (GAE) per 100 g.

#### Total alkaloid content

Alkaloids are known for their diverse pharmacological activities, including anti-inflammatory effects. TAC analysis helps assess the concentration of alkaloids, providing information on their potential contribution to the anti-arthritic properties of the substance. To determine the alkaloid content in a sample, 0.625 g of the sample were combined with 200 ml of a solution containing 10% acetic acid in ethanol. This mixture was left to stand for 4 h and then heated until it reduced to one-quarter of its original volume. Following this, the solution was filtered, and 15 drops of concentrated ammonium hydroxide were introduced. After allowing the precipitate to form for 3 h, the supernatant (the liquid above the precipitate) was removed. The remaining precipitate was washed using a 0.1M ammonium hydroxide solution and then filtered to collect the residue. Finally, the weight of this residue was measured to determine the alkaloid content in the sample [38].

$$\%\;\text{Alkaloid}=\frac{\text{Weight}\;\text{of}\;\text{alkaloid}}{\text{Weight}\;\text{of}\;\text{sample}}\times 100$$

#### Total steroid content

Steroids are known for their anti-inflammatory and immunomodulatory effects. Determining TSC helps gauge the presence of steroids in the sample, which may contribute to the anti-arthritic activity. One milliliter of test sample was mixed with 4N sulphuric acid, 0.5% iron chloride and potassium hexacyanoferrate solution, heated at 72°C and diluted with distilled water, and the absorbance was measured at 780 nm [39].

#### Total tannin content

Tannins exhibit anti-inflammatory and antioxidant properties. TTC analysis quantifies the tannin concentration, providing information on their potential role in mitigating arthritis-related inflammation. The tannin content in a sample extract was assessed using the Folin-Ciocalteu method. About 0.1 ml of the sample extract were mixed with 7.5 ml of distilled water, 0.5 ml of Folin Ciocalteu phenol reagent and 1 ml of a 35% sodium carbonate solution in a 10-ml volumetric flask. The total volume was adjusted to 10 ml with distilled water. After agitation, the mixture stood at room temperature for 30 min. Reference standard solutions of tannic acid were prepared, and their absorbance at 700 nm was measured using a UV/Visible spectrophotometer, with a blank as a reference. Tannin content was determined in triplicate, and the results were expressed in milligrams of tannic acid equivalents per gram of dried sample [40].

### *In vitro* anitioxidant activity

#### DPPH (2,2-diphenyl-1-picrylhydrazyl) assay

Arthritis is often associated with oxidative stress. *In vitro* antioxidant activity assessment helps understand the substance's ability to neutralize free radicals, potentially alleviating oxidative stress associated with arthritis. Prepare a 0.1 mM DPPH solution in methanol by dissolving 0.0216 g of DPPH in 1 liter of methanol. Mix 100 μl of the 0.1 mM DPPH solution with 300 μl of the sample solution at different level of concentrations (500, 250, 100, 50 and 10 μg/ml). Vigorously shake the mixture for thorough mixing and let it sit at ambient temperature for half an hour. During this period, the antioxidants in the sample will react with the DPPH radical, causing a color change from purple to yellow. After the 30-min incubation, measure the absorbance of the solutions at 517 nm using a UV-VIS spectrophotometer. Ascorbic acid at a concentration of 1 mg/ml serves as the reference standard. You can evaluate the antioxidant potential of your sample by comparing its scavenging activity to that of ascorbic acid, with lower absorbance values indicating higher free radical scavenging activity. The scavenging capability of the DPPH radical can be calculated using a specified formula [41].

$$\begin{array}{l} \text{DPPH}\;\text{scavenging}\;\text{effect}(\%\;\text{inhibition})=\\ \left[\text{absorbance}\;\text{of}\;\text{control}\;(\text{absorbance}\;\text{of}\;\text{control}-\text{absorbance}\;\text{of}\;\text{reaction}\;\text{mixture})\right] \end{array}\times 100$$

#### ABTS (2,2′-azino-bis(3-ethylbenzothiazoline-6-sulfonic acid)) assay

The modified ABTS assay involved preparing stock solutions of 7 mM ABTS and 2.4 mM potassium persulfate. The working solution resulted from equal volume mixing of these stock solutions allowing them to react for 14 h at room temperature, shielded from light. This working solution was then diluted by combining 1 ml of the ABTS solution with 60 ml of methanol until the absorbance reached 0.706 ± 0.01 units at 734 nm, measured with a spectrophotometer. For each assay, a fresh ABTS solution was prepared, and different sample concentrations (500, 250, 100, 50 and 10 μg/ml) were allowed to react with 1 ml of the ABTS solution. After 7 min, the absorbance of these mixtures was measured at 734 nm using a spectrophotometer. The ABTS scavenging capacity of the sample was compared with that of ascorbic acid, a standard reference. The percentage inhibition or ABTS radical scavenging activity was calculated using a specified formula.

$$\text{ABTS}\;\text{radical}\;\text{scavenging}\;\text{activity}\;(\%)=\frac{\left(\text{Abs}\_\text{control}-\text{Abs}\_\text{sample}\right)}{\text{Abs}\_\text{control}}$$

Where, Abs_control is the absorbance of the ABTS radical in methanol without the sample.

Abs_sample is the absorbance of the ABTS radical solution mixed with the sample extract or standard.

All measurements and assays were carried out in triplicate (n = 3) to ensure accuracy and consistency) [42].

#### FRAP (ferric reducing antioxidant power) assay

The FRAP assay was conducted using Roche Diagnostic Systems, Inc. (NJ, USA) COBAS-FARA (Software version 8529) centrifugal fast analyzer. A total of 300 μl of freshly prepared FRAP reagent was heated to 37°C. Initially, a blank reading was taken at 593 nm using a spectrophotometer. Samples were added at various concentrations, along with water (H2O), resulting in a final dilution ratio of 1/34. Absorbance readings were recorded at 593 nm at regular 15-s intervals throughout the monitoring period. To determine the change in absorbance at 593 nm, the final reading was subtracted from the initial M1 reading for each sample. This alteration in absorbance at 593 nm offers insights into the antioxidant capacity of the samples, where a greater change indicates higher antioxidant activity. The FRAP assay assesses the ability of antioxidants to reduce ferric ions to ferrous ions, serving as an indicator of their antioxidant potential [43].

#### Nitric oxide assay

The process began by preparing extracts from an initial crude extract concentration of 50 mg/ml. These extracts were subsequently mixed with dimethyl sulfoxide to create a range of concentrations from 500 to 10 μg/ml. The resulting solutions were stored at 4 °C for future use. For each sample, 150 μl of the extract was mixed with an equal volume of freshly prepared Griess reagent. Similar control samples were prepared without the extracts, containing an equal volume of buffer to mirror the test samples' procedure. After a 30-min incubation, transfer 100 μl of the reaction mixture into a 96-well plate. Measure the sample's absorbance at 540 nm using a UV-Vis microplate reader, specifically a Molecular Devices instrument in Georgia, USA. This procedure is likely related to an assay measuring nitric oxide levels, as the Griess reagent is commonly used to quantify nitrite, a stable breakdown product of nitric oxide. The change in absorbance at 540 nm indicates the presence and concentration of nitrite, relevant to various biological and chemical processes [44].

$$\text{Nitic}\;\text{Oxide}\;\text{Scavenge}\;(\%)=\frac{{\text{A}}_{\text{control}}-{\text{A}}_{\text{test}}}{{\text{A}}_{\text{control}}}$$

The limitation of nitric oxide (NO) inhibition as a marker of anti-inflammatory properties lies in the dual role of NO in inflammation. While excessive NO production can contribute to inflammation, NO also plays a crucial role in the resolution of inflammation and tissue repair. Overly aggressive inhibition of NO may hinder its beneficial functions, such as vasodilation, immune response modulation and antimicrobial activity. This duality complicates the straightforward interpretation of NO inhibition as a measure of anti-inflammatory efficacy. A balanced approach is needed to avoid potential unintended consequences associated with the indiscriminate suppression of NO.

### *In vitro* anti-arthritic activity

#### Protein denaturation assay

Arthritis involves inflammatory processes that can lead to the denaturation of proteins, contributing to tissue damage and exacerbating the condition. By assessing the ability of the substance to inhibit protein denaturation, researchers aim to understand its potential in preventing or mitigating the protein structural changes associated with arthritis. A positive result in this assay suggests a protective effect against inflammation-induced protein damage. The primary cause of inflammation is the modification of protein structures. To assess the potential to prevent protein denaturation different concentrations (500, 250, 100, 50 and 10 μg/ml) of the test extract were mixed with 500 μl of a 1% bovine serum albumin solution. The mixture stood at room temperature for 10 min and was then heated at 51 °C for 20 min. After the heating process, the solution was allowed to cool down to ambient temperature, and the absorbance was determined at 660 nm. Acetyl salicylic acid served as the positive control for reference. The experiment was replicated three-times to ensure precision, and the percentage of protein denaturation inhibition was computed using a designated formula.

$$\%\;\text{Inhibition}=100-\left(\frac{\left(\text{A}1-\text{A}2\right)}{\text{A}0}\right)\times 100$$

Where, A1 is the absorbance of the control, A2 is the absorbance of the test sample, A0 is the absorbance of the positive control. Additionally, a dose-response curve was created to find the IC50 values, representing the concentration needed for a 50% maximum protective effect. All tests and analyses were performed three-times and averaged to ensure dependable results [45].

#### Proteinase inhibition assay

Proteinases, including enzymes like proteases, play a role in the breakdown of proteins, contributing to tissue degradation in arthritis. Evaluating the substance's ability to inhibit proteinases provides insights into its anti-arthritic potential by assessing its capacity to hinder the enzymatic processes involved in tissue degradation. A positive outcome in this assay indicates a potential protective effect against proteinase-mediated damage. The proteinase inhibitory assay, modified from Oyedepo and Femurewa's method, involved creating a reaction mixture of 2 ml with 0.06 mg trypsin, 1 ml Tris-HCl buffer (20 mM, pH 7.4), and 1 ml of the test plant extract (HO) at various concentrations. After incubating at 37 °C for 5 min, 1 ml of 0.8% (w/v) casein was added, and the mixture incubated for an additional 20 min. To stop the reaction, 2 ml of 70% perchloric acid was added. After centrifugation, the absorbance of the supernatant was measured at 210 nm, using a Tris-HCl buffer as a blank. The entire experiment was conducted in triplicate for result reliability [46].

### Gas chromatography & mas spectroscopy

The GC-MS analysis of *Abrus precatorius* macerated oil was conducted to identify and characterize its chemical constituents. This analytical technique allows for the separation and identification of individual compounds within the oil, providing a detailed profile of its chemical composition. The macerated oil from the aerial parts of *Abrus precatorius* underwent analysis using gas chromatography and mass spectrometry (GC-MS) with a Clarus 680 GC system. A fused silica column (Elite-5MS) was utilized, with dimensions of 30 m in length, 0.25 mm in internal diameter and a film thickness of 250 μm, using helium as the carrier gas. The injector temperature was set at 260 °C, and a 1 μl sample of the extract followed a programmed temperature sequence: starting at 60 °C for 2 min, increasing to 300 °C at a rate of 10 °C per minute, and holding at 300 °C for 6 min. The mass detector conditions involved maintaining transfer line and ion source temperatures at 240 °C, with an electron impact ionization mode at 70 eV. Mass spectra were generated within the range of 40–600 Da. To identify compounds in the macerated oil, mass spectra were compared with a database in the GC-MS system, enabling the characterization of individual constituents [47,48].

### *In silico* molecular docking

Subsequently, *in silico* molecular modeling was performed on the constituents identified through GC-MS analysis. *In silico* modeling involves the use of computational techniques to simulate and analyze molecular interactions. This approach allows researchers to predict the potential bioactivity and binding affinities of the identified compounds with molecular targets relevant to anti-arthritic effects. Protein structures was obtained from RCSB Protein Data Bank in pdb format. Discovery Studio provides a user-friendly interface to easily import and preprocess protein molecules. Protein preparation by discovery studio involves the removal of water molecules, addition of hydrogen atoms, and assignment of atom types and charges. This helps in ensuring that the protein is in the correct conformation and ready for docking experiments. Additionally, Discovery Studio allows for the removal of unwanted protein chains or residues, enabling researchers to focus on specific regions of interest. The software also offers advanced visualization tools to analyze the protein's active site and identify potential binding pockets for the ligand. The structures of the GCMS ligands were retrieved from Pubchem in sdf format and converted to pdb format using Open Babel. The ligands were then further prepared by assigning proper bond orders and adding hydrogen atoms using the AutoDock Tools software. The protein and ligand files were saved in pdbqt format for docking simulations. To enhance interactions, polar hydrogens were added to the protein during preparation, followed by Kollman calculations and Gasteiger charge determination. The simulations were executed with a grid box defined around the active site of the protein, allowing for optimal docking of the ligands. The docking simulations were performed using AutoDock Vina software, which utilizes a scoring function to predict the binding affinity between the protein and ligands. The docking results were analyzed for binding energies and interactions between ligands and proteins. This provided insights into potential binding modes and affinities. Discovery Studio was utilized to visualize 2D and 3D structures [49].

### Statistical analysis

Each experiment was carried out in triplicates and the data was expressed as mean ± standard deviation and concentrations were determined in micrograms/milliliters, respectively.

## Results

### Preliminary phytochemical screening

Various phytochemical tests were carried out to detect the presence of phytoconstituents in the oil. The phytochemical screening of the oil uncovered the presence of carbohydrates, flavanoids, saponins, gums and alkaloids as shown in Table 1, while carboxylic acids, tannins, steroids, glycosides, proteins, phenols, biuret and flavanoglycosides were absent.

**Table 1.** Preliminary phytochemical screening tests of *Abrus precatorius* macerated oil.

S.No.	Name of the sample	Phytochemical compound	Result
1.	Macerated oil	Resins	+
2.		Carboxylic acid	–
3.		Tanins	–
4.		Steroids	–
5.		Flavonoid	+
6.		Carbohydrates	+
7.		Glycosides	–
8.		Saponification	–
9.		Protein	–
10.		Phenol	–
11.		Biuret	–
12.		Saponin	+
13.		Gum	+
14.		Flavanoglycosides	–
15.		Alkaloids	+

+ Indicated presence and – indicates absence.

### Quantitative estimation of phytoconstituents

**Figure 2.**
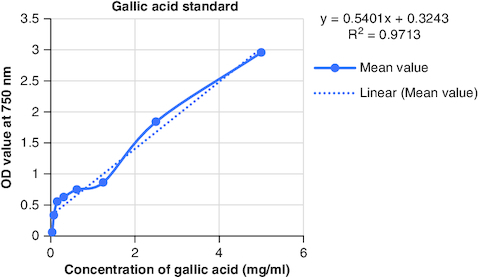
Calibration curve of gallic acid at various concentrations.

**Figure 3.**
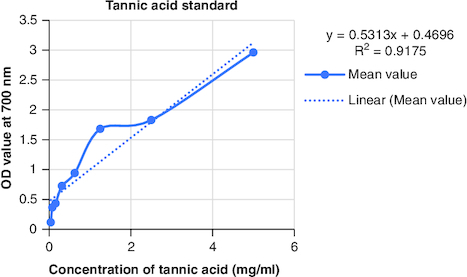
Calibration curve of tannic acid at various concentrations.

**Figure 4.**
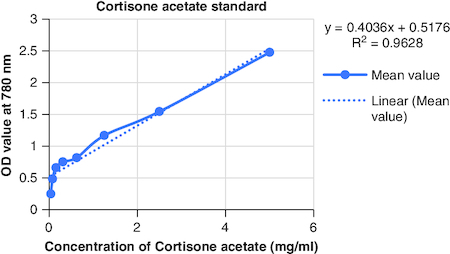
Calibration curve of cortisone acetate at various concentrations.

The macerated oil's phytochemicals were quantitatively assessed. Total phenolic content was determined using the Folin–Ciocalteu method with gallic acid as the reference, yielding a content of 0.2061 mg GAE/g. The calibrartion curve of gallic acid is shown in Figure 2. The total alkaloid content was 35.2% for 0.625 g of the oil. Total tannin content was estimated as 0.2040 mg TAE/g of tannic acid using the Folin–Ciocalteu method and tannic acid as the reference. The calibrartion curve of tannic acid is shown in as depicted in Figure 3. The total steroid content, specifically cortisone acetate, was found to be 0.257 mg per ml of oil, with the calculation based on a calibration curve shown in Figure 4, using cortisone acetate as the standard at various concentrations.

### *In vitro* antioxidant activity

**Figure 5.**
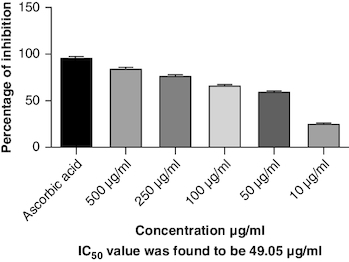
DPPH scavenging activity.

**Figure 6.**
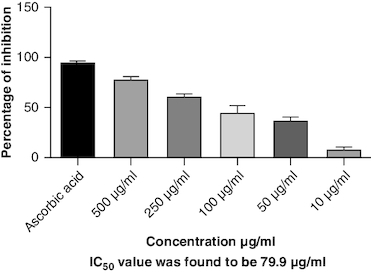
ABTS scavenging activity.

**Figure 7.**
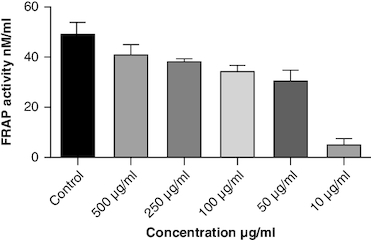
Ferric reducing activity of macerated oil.

**Figure 8.**
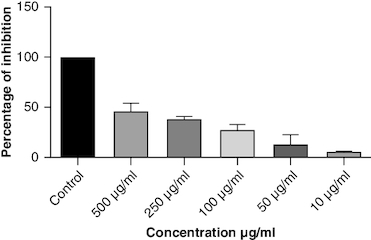
Nitric oxide scavenging activity.

DPPH (2,2-diphenyl-1-picrylhydrazyl) is a stable free radical frequently employed to gauge the antioxidant capability of a substance. The macerated oil was tested at different concentrations in this assay, and the inhibition percentage of DPPH was determined. Ascorbic acid, a recognized antioxidant, served as a reference standard, showing significant inhibition, with an average around 96.42%. The macerated oil demonstrated significant antioxidant activity. At a concentration of 500 μg/ml, it displayed an inhibition rate of 84.68%, indicating its ability to scavenge free radicals as shown in Figure 5. The IC_50_ value of 49.05 suggests that the concentration at which the oil inhibits 50% of the DPPH radicals is relatively low, signifying potent antioxidant potential. ABTS (2,2′-azino-bis(3-ethylbenzothiazoline-6-sulfonic acid)) is a common method to evaluate antioxidant activity, assessing the macerated oil's capability to reduce ABTS radicals. Similar to the DPPH assay, ascorbic acid exhibited strong inhibition, averaging around 95.20%. The highest DPPH scavenging activity was observed in ascorbic acid, a potent antioxidant, with an IC_50_ value of 49.05 μg/ml. The macerated oil showed notable ABTS scavenging activity, mentioned in Figure 6. At a concentration of 500 μg/ml, it displayed an inhibition rate of 78.13%. The IC_50_ value of 79.94 suggests that the oil is effective at neutralizing ABTS radicals at a concentration close to the reference standard. IC50 determination for both the assays involved the use of the log(inhibitor) versus normalized response model with a variable slope for dose-response curve fitting, as implemented in GraphPad Prism. The FRAP assay measures the reducing capacity of the macerated oil, which is an indicator of its ability to donate electrons and neutralize free radicals. The control (without the oil) had a mean FRAP value of 49.46, indicating the baseline. The oil, at concentrations spanning from 10 μg/ml to 500 μg/ml, exhibited a rise in FRAP activity that is dependent on concentration, as illustrated in Figure 7. This outcome indicates the oil's capacity to decrease iron and implies its potential as an antioxidant. Nitric oxide (NO) plays a role in diverse physiological processes but can be detrimental when excessively produced. The test gauges the oil's capability to block the production of NO. At 500 μg/ml concentration, the oil demonstrated notable NO inhibition (46.26%), pointing to potential anti-inflammatory properties, as depicted in Figure 8. While the IC50 value is not given, the concentration-dependent response implies the oil is effective in inhibiting NO production.

### *In-vitro* antiarthritic activity

**Figure 9.**
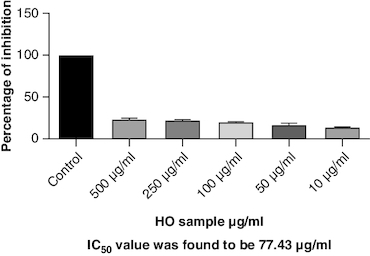
Albumin denaturation assay.

**Figure 10.**
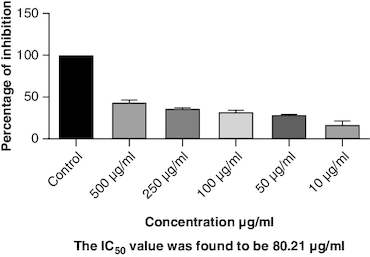
Proteinase inhibition assay.

Protein denaturation is a process where the 3D structure of a protein is disrupted, often leading to loss of its functional properties. Inhibition of this denaturation process can be a sign of potential anti-inflammatory or cytoprotective properties of a substance. The control group represents the protein denaturation percentage. The macerated oil, at 500 μg/ml concentration, strongly prevented albumin denaturation, showing a mean inhibition of 23.81%. The IC_50_ value of 77.43 indicates the concentration at which the oil inhibits 50% of albumin denaturation, demonstrating its potential as an anti-inflammatory agent. The results are represented graphically in Figure 9. Proteinases, also known as proteases, are enzymes that break down proteins. The macerated oil, at various concentrations, displayed a concentration-dependent inhibition of proteinase activity. At 500 μg/ml, it exhibited a mean inhibition of 43.96% as in Figure 10. The IC_50_ value of 80.21 indicates the concentration at which the oil inhibits 50% of proteinase enzyme activity, highlighting its potential as an anti-proteolytic agent.

### GC-MS

**Figure 11.**
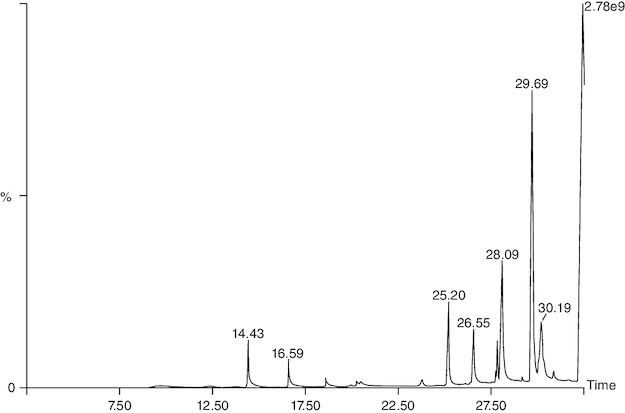
Chromatogram of macerated oil using gas chromatography and mass spectroscopy.

Gas chromatography-mass spectrometry (GC-MS) analysis identified various compounds in the macerated oil, including N-Hexadecanoic acid, T-Butyl cyclopentaneperoxycarboxylate, octadecane, octanoyl chloride, octanoic acid, myristic anhydride and tetracosane. These compounds may contribute to the pharmacological activity of oil against arthritis. The compounds, molecular formula, molecular weight, retention time is mentioned in Table 2 & Figure 11.

**Table 2.** Phytoconstituents detected in macerated oil using gas chromatography and mass-spectroscopy.

S.NO	Retention time	Compound name	Molecular weight	Molecular formula
1	14.43	N-Hexadecenoic acid	256	C_16_H_32_O_2_
2	16.59	T-Butyl Cyclopentaneperoxycarboxylate	186	C_10_H_18_O_3_
3	25.20	Octadecane	254	C_18_H_38_
4	26.54	Octanoyl Chloride	162	C_8_H_15_OCl
5	29.68	Octanoic acid	316	C_14_H_18_O_2_BrF
6	30.19	Myristic anhydride	438	C_16_H_30_O_2_
7	32.40	Tetracosane	338	C_24_H_50_

### *In silico* studies

In the molecular docking studies involving bioactive compounds from *Abrus precatorius* macerated oil, the binding energies against two important protein targets, TNF α and IL-6, were assessed. In Table 3, the compounds were docked against TNF α, and the results revealed varying binding energies. Notably, Myristic anhydride exhibited the strongest binding affinity with a score of -8.6 Kcal/mol, followed by T-Butyl Cyclopentaneperoxycarboxylate and octanoic acid with binding energies of -7.5 Kcal/mol and -7.7 Kcal/mol, respectively, is shown in Figure 12. The same set of compounds were docked against IL-6, demonstrating different binding affinities. Octanoic acid showed the highest binding energy of -6.1 Kcal/mol, followed by myristic anhydride with a score of -5.6 Kcal/mol, and is shown in Figure 13. It is important to note that the binding affinities varied for the same compounds when interacting with different protein targets, highlighting their potential selectivity and specificity in modulating these key proteins associated with inflammation and immune responses [50].

**Table 3.** Molecular docking scores of bioactive compounds from *Abrus precatorius* macerated oil against TNF α and IL 6.

S.no	Ligands	Binding energy TNF- α (Kcal/mol)	Binding energy IL-6 (Kcal/mol)
1	T-Butyl cyclopentaneperoxycarboxylate	-7.5	-6.3
2	Octadecane	-6.5	-5.3
3	Octanoyl chloride	-6.6	-6.7
4	Octanoic acid	-7.7	-6.1
5	Myristic anhydride	-8.6	-5.6
6	Tetracosane	-6.9	-4.2

Figure 12.Bioactive compounds present in *Abrus precatorius* macerated oil docked with TNF -α (2AZ5).

**Figure:**
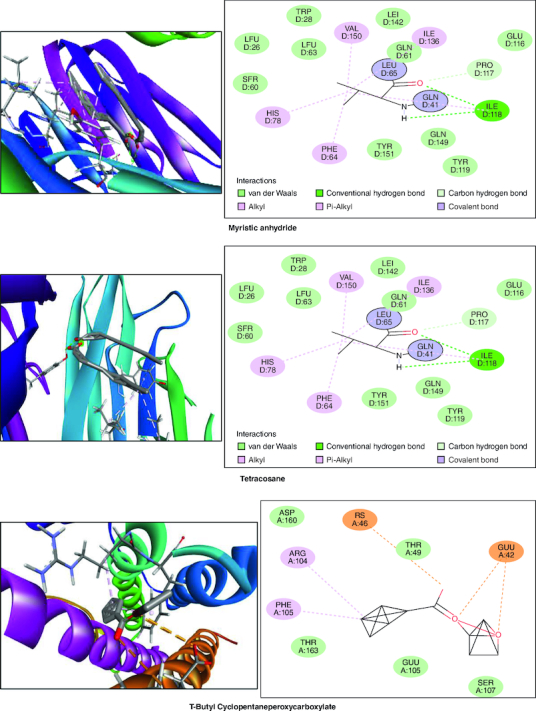

**Figure:**
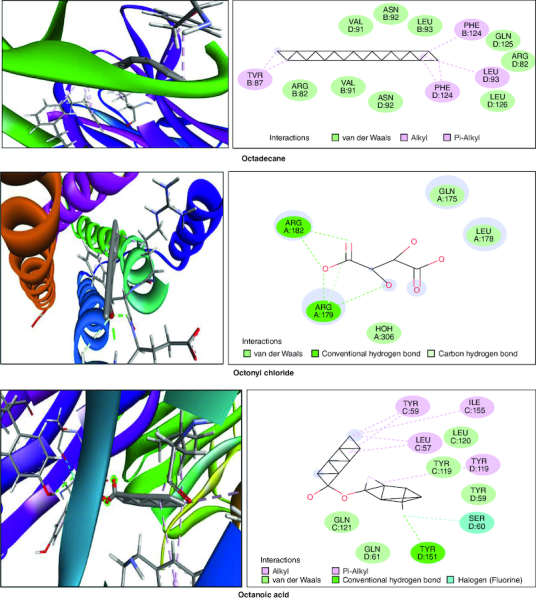

Figure 13.Bioactive compounds present in *Abrus precatorius* macerated oil docked with IL-6 (1ALU).

**Figure:**
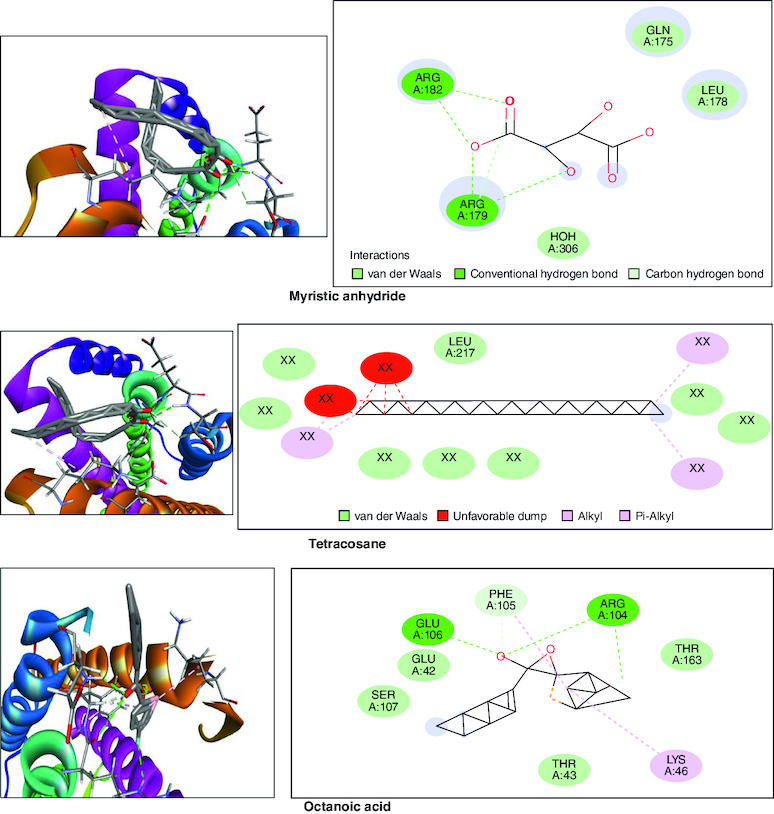

**Figure:**
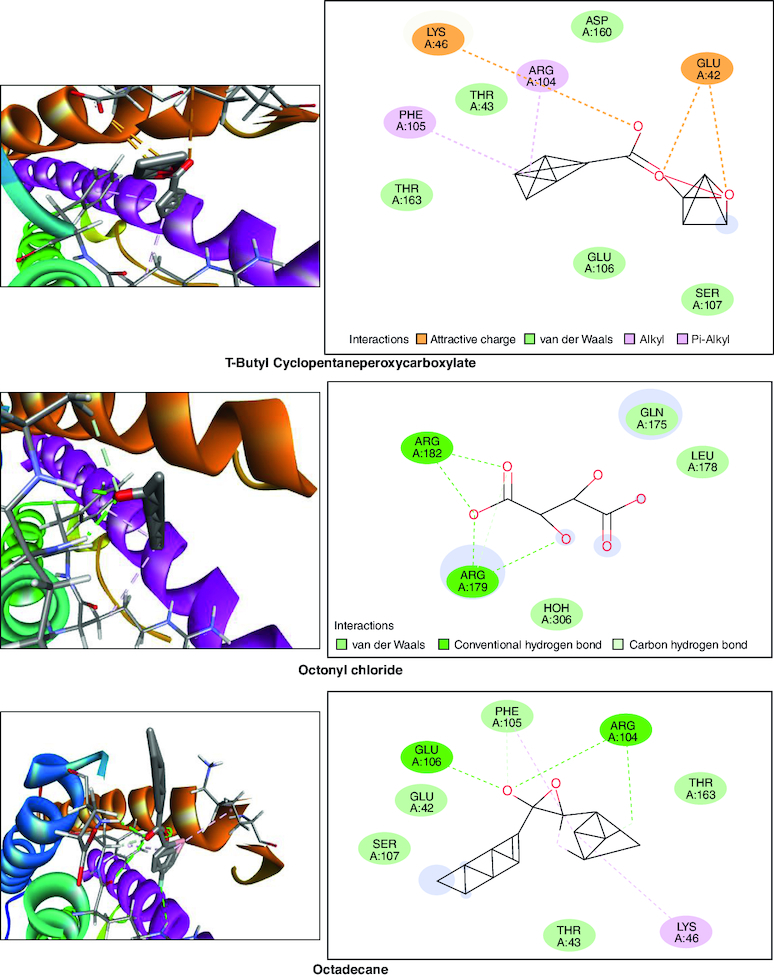

## Discussion

In recent years, there has been growing interest in the potential therapeutic benefits of natural products for the treatment of arthritis. *Abrus precatorius*, commonly known as the rosary pea, is a plant that has been traditionally used in folk medicine for its anti-inflammatory properties [51]. In this study, we aimed to evaluate the anti-arthritic activity of macerated oil extracted from *Abrus precatorius* and investigate its potential as a natural remedy for arthritis

The oil of aerial parts *of Abrus precatorius* has been used in many parts of India, mainly southern India for the treatment of body pains, swellings, etc. Leaves of the plant are mixed in warm oil and applied for relief from rheumatic pain [52]. The oil used in the study has been prepared by the maceration of the powdered drug in coconut oil. Studies have reported that coconut oil also posesses anti-inflammatory, analgesic properties [53]. Various chemical tests were conducted to assess the phytochemical composition of the prepared oil. Phytochemical screening is a qualitative analysis of plant extracts to identify phytochemicals, aiding in drug discovery and assessing herbal product quality and potency [54]. It revealed the presence of gums, resins, carbohydrates, alkaloids and saponins. Quantitative estimation of the oil revealed the presence of alkaloids, tannins, steroids and phenols in minor quantities. Phenolic compounds are known for their ability to neutralize harmful free radicals in the body, which can have various health benefits, such as reducing the risk of chronic diseases. The existence of alkaloids implies that the macerated oil may have various biological effects, including analgesic, anti-inflammatory or antimicrobial properties. Similarly, steroids also exhibit anti-inflammatory and immunomodulatory effects [55].

Oxidative stress occurs when the body produces too many reactive oxygen species (ROS) or lacks antioxidants, which can damage cells, tissues and DNA [56]. The inflammation resulting from oxidative stress, which harms cells and tissues, triggers transcription factors like NF-κB, and elevates the generation of reactive oxygen species (ROS) [57]. This inflammatory process can contribute to diverse chronic conditions, such as arthritis [58]. Antioxidants have been found to have protective effects against tissue damage, potentially leading to clinical improvement in patients [59]. In this study, the oil's antioxidant activity was evaluated using DPPH, ABTS, FRAP and NO assays. The DPPH assay gauges the extract's capacity to transform DPPH-free radicals into nonradical diphenyl picryl hydrazine, resulting in a color change from purple to yellow [60]. Macerated oil was compared with the standard antioxidant ascorbic acid, which exhibited dose-dependent antioxidant activity in DPPH assay. The IC _50_ value of the oil was found to be 49.08 μg/ml. Antioxidant activity and IC _50_ value is inversely proportional. The oil showed maximum percentage of inhibition at 500 μg/ml (84.68%), in comparison with standard ascorbic acid 96.41% at the same concentration, whose results are in accordance with the previous study [61]. These findings indicate that the oil has some antioxidant potential, but it is not as effective as ascorbic acid in neutralizing DPPH-free radicals. Ascorbic acid was kept as a reference standard in our study based on its well-established and widely recognized use in similar studies within our research field [62,63]. Ascorbic acid serves as a benchmark due to its stability, reproducibility and availability, making it a reliable reference for comparative analysis. Additionally, its inclusion allows for a more comprehensive evaluation of the experimental results. Additionally, we conducted the ABTS assay, which relies on antioxidants causing a color change in the ABTS radical cation (ABTS•+) [64]. The IC50 value for the oil in the ABTS assay was determined to be 79.94 μg/ml. In the FRAP assay, antioxidants are tested for their ability to change Fe3+ to Fe2+ in the presence of tripyridyltriazine (TPTZ), forming a strong blue Fe2+–TPTZ complex [65]. NO can contribute to joint inflammation and damage by promoting the production of pro-inflammatory cytokines and by damaging cartilage and other joint tissues [66]. The oil showed concentration-dependent NO inhibition whose results are in accordance with a previous study [67].

Protein denaturation refers to the alteration in the structure and function of proteins, which can be triggered by various factors including oxidative stress and inflammation. In the context of arthritis and inflammation, protein denaturation has been observed to play a crucial role in the progression and severity of joint-related disorders. Exploring the connection between protein denaturation and joint inflammation could offer valuable insights for developing specific treatments for arthritis and related conditions. The present study exhibited a concentration-dependent inhibition of protein denaturation throughout the concentration range 10–500 μg/ml. The macerated oil exhibited 23% of inhibition at 500 μg/ml. These results are in concordance with previously carried out study [68].

Proteinase inhibitors have been found to be beneficial in the treatment of arthritis. These inhibitors work by binding to proteases and preventing them from degrading proteins, thus preserving the integrity and function of tissues [69]. Additionally, proteinase inhibitors play a role in regulating important biological processes such as blood clotting, immune response and cell growth. Without proteinase inhibitors, excessive protease activity can lead to tissue damage and inflammation. By inhibiting the activity of proteases involved in joint degradation, proteinase inhibitors can help to reduce inflammation, maintain joint integrity and alleviate the symptoms of arthritis [70]. The macerated oil showed 43% of inhibition when compared with control at 500 μg/ml.

In the GC-MS analysis, various compounds were identified in the macerated oil, including N-Hexadecanoic acid, Octadecane and Tetracosane, each with distinct retention times, molecular weights and formulas. The presence of these bioactive compounds indicates the existence of fatty acids and long-chain hydrocarbons in the oil. Fatty acids are recognized for their anti-inflammatory properties, which are pertinent to arthritis. The molecular docking results shed light on the potential binding affinities of these compounds with key inflammatory targets involved in arthritis, specifically TNF-α and IL-6. Notably, dodecanoic acid, 1-(hydroxymethyl)-1,2-ethanediyl ester and myristic anhydride displayed the most favorable binding energies with TNF-α, suggesting their strong potential to modulate this pro-inflammatory cytokine. Furthermore, N-hexadecanoic acid, octanoyl chloride and octanoic acid also exhibited notable binding affinities with TNF-α, indicating their possible anti-inflammatory effects.

TNF-α and IL-6 are pivotal cytokines with essential roles in the pathophysiology of RA. These cytokines are generated by immune cells, such as macrophages and T cells. In RA, its levels are significantly elevated in the synovium. TNF-α stimulates osteoclasts, cells responsible for bone resorption which leads to joint erosion and destruction of cartilage and also contributes to synovial hyperplasia, causing the synovium to thicken and invade the joint space, further promoting joint damage [71]. IL-6 is a pleiotropic cytokine which causes the production of C-reactive protein (CRP) and other inflammatory markers in the blood. Elevated CRP levels are often seen in RA patients [72].

IL-6 is significantly involved in stimulating B cells, resulting in the generation of autoantibodies like RA and anti-citrullinated protein antibodies (ACPAs), which are distinctive features of RA. It also contributes to synovial hyperplasia by promoting the survival and proliferation of fibroblast-like synoviocytes [73]. TNF-α and IL-6 are important contributors to inflammation. They trigger different signaling pathways like NF-κB, JAK-STAT and MAPK, leading to the production of inflammatory molecules that sustain the inflammatory response in RA [74]. These results collectively underscore the pharmacological potential of *Abrus precatorius* macerated oil as a source of bioactive compounds with anti-arthritic properties.

## Conclusion

This study presents evidence supporting the potential anti-arthritic effects of macerated oil derived from *Abrus precatorius*, likely attributed to its anti-inflammatory and antioxidant properties. Future investigations should delve into the mechanisms of action and determine the optimal therapeutic dosage. A comprehensive safety assessment through toxicological studies is imperative for prolonged use. Larger clinical trials involving diverse populations are necessary to validate the oil's efficacy in arthritis treatment. If proven both effective and safe, *Abrus precatorius* macerated oil could emerge as a promising alternative to conventional medications, minimizing reliance on synthetic drugs and their potential side effects. This research contributes to the expanding knowledge base on natural remedies for arthritis management, offering hope for those exploring alternative treatments for this challenging condition.
